# A novel micronemal protein MP38 is involved in the invasion of merozoites into erythrocytes

**DOI:** 10.1128/mbio.03917-24

**Published:** 2025-04-09

**Authors:** Tuyet-Kha Nguyen, Sy-Thau Nguyen, Van-Truong Nguyen, Sung-Hun Na, Robert W. Moon, Jetsumon Sattabongkot, Yee Ling Lau, Won-Sun Park, Wan-Joo Chun, Feng Lu, Seong-Kyun Lee, Jin-Hee Han, Eun-Taek Han

**Affiliations:** 1 Department of Medical Environmental Biology and Tropical Medicine, Kangwon National University School of Medicine 85082, Chuncheon-si, Gangwon-do, South Korea; 2 Institue of Clinical Infectious Diseases, 108 Military Central Hospital, Hanoi, Vietnam; 3 Department of Obstetrics and Gynecology, Kangwon National University School of Medicine 85082, Chuncheon-si, Gangwon-do, South Korea; 4 Department of Infection Biology, Faculty of Infectious and Tropical Diseases, London School of Hygiene and Tropical Medicine 218289, London, England, United Kingdom; 5 Mahidol Vivax Research Unit, Faculty of Tropical Medicine, Mahidol University 115374, Bangkok, Thailand; 6 Department of Parasitology, Faculty of Medicine, Universiti Malaya 65300, Kuala Lumpur, Malaysia; 7 Department of Physiology, School of Medicine, Kangwon National University 85082, Chuncheon-si, Gangwon-do, South Korea; 8 Department of Pharmacology, School of Medicine, Kangwon National University 85082, Chuncheon-si, Gangwon-do, South Korea; 9 Department of Pathogen Biology and Immunology, School of Medicine, Yangzhou University 74544, Yangzhou, Jiangsu, China; Washington University in St. Louis School of Medicine, St. Louis, Missouri, USA; National Institute of Allergy and Infectious Diseases, Rockville, Maryland, USA

**Keywords:** malaria, *Plasmodium vivax*, PvMP38, micronemal protein, complex formation, CRISPR/Cas9

## Abstract

The absence of an *in vitro* cultivation system for *Plasmodium vivax* hinders the exploration of molecular targets for vaccine development. To address this, functional studies often rely on alternative models, such as *P. knowlesi*, due to its genetic similarity to *P. vivax*. This study investigated the role of a novel micronemal protein, PvMP38 (PVX_110945), in both *P. vivax* and *P. knowlesi* merozoite invasion of erythrocytes. The full-length ectodomain of PvMP38 was expressed, and polyclonal antibodies were generated to assess its function. PvMP38 was confirmed to localize on micronemal organelle in both *P. vivax* and *P. knowlesi* merozoites. *In vitro* protein-protein interaction assays revealed that PvMP38 binds to Pv12 with high-affinity interaction. A conserved novel complex of Pv12-Pv41-PvMP38 was identified by immunoprecipitation of *P. vivax* antibodies on *P. knowlesi* schizont lysates. Linear epitopes of PvMP38 with high and moderate antigenicity were identified in clinical isolates of both species. Invasion inhibition assays demonstrated that a triple antibody combination targeting the PvMP38, Pv12, and Pv41 significantly reduced *P. knowlesi* merozoite invasion of erythrocytes compared to a single antibody. In addition, CRISPR/Cas9-mediated knockout of *P. knowlesi mp38* markedly impaired parasite growth, underscoring its essential role during the asexual stage. These findings identify PvMP38 and its associated complex as promising targets for malaria interventions and highlight the utility of *P. knowlesi* as a model for investigating *P. vivax* erythrocyte invasion mechanisms.

**IMPORTANCE:**

This manuscript reported an effort in malaria eradication by identifying and functionally characterizing a novel *Plasmodium vivax* micronemal protein, PvMP38, involved in erythrocyte invasion. A narrow repertoire of an efficacious vaccine targeting *P. vivax* candidates is being developed due to the lack of continuous *in vitro* culture. This study addresses a gap in *P. vivax* research using *P. knowlesi* as a model for both genome editing and antibody functionality validation. By enhancing the protein-protein interaction screening framework, this study demonstrated that PvMP38 forms a complex with Pv12 and Pv41, opening the approaches to multi-antigen vaccines. The successful application of CRISPR/Cas9 gene editing techniques to disrupt its homolog, the *pkmp38* gene, further assesses the protein’s significance in the growth and invasion of the parasite. These findings provided valuable insights into the biology of *P. vivax* and proposed PvMP38 as a promising candidate for malaria intervention strategies.

## INTRODUCTION

Among the five *Plasmodium* species that infect humans, *P. vi*vax is a significant cause of morbidity, particularly in Southeast Asia. Most *P. vivax*-related mortality occurs during the asexual blood stage, underscoring the need for developing vaccines targeting this stage (1). Current vaccine efforts have focused on mimicking natural immunity to inhibit parasite invasion and erythrocyte development (2, 3). Despite *P. vivax* Duffy-binding protein region II (PvDBPII) showing heterologous protection in Phase I/IIa clinical trials, vaccines targeting multiple *dbpII* alleles elicit higher responses than a single allele because of the high polymorphic properties (4, 5). Other studies also have indicated that single antigens induce protection against the homologous but not heterologous strains (6, 7). Therefore, a systematic approach to investigate additional candidates is needed to understand the *P. vivax* invasion mechanism and increase the chance of generating effective multivalent blood-stage vaccines.

The paucity of vaccine candidates characterized, particularly for *P. vivax*, is a consequence of several technical obstacles: (i) *P. vivax* only invades reticulocytes, limiting continuous *in vitro* cultivation (8), (ii) difficulties in expressing membrane-bound proteins in soluble form, and (iii) sensitivity limitations in detecting low-affinity protein-protein interaction (PPI) hinder the exploration of a new blood-stage candidate (9–11). These challenges necessitate the use of alternative models to study their invasion mechanisms. *P. knowlesi*, a zoonotic parasite with a close phylogenetic relationship and antigen cross-reactivity with *P. vivax* (12, 13). Unlike *P. vivax*, *P. knowlesi* can be cultivated in human erythrocytes *in vitro*, providing the opportunity to explore the invasion mechanisms (14). The advance of clustered regularly interspaced palindromic repeats (CRISPR)-Cas9 genomic modification in *P. knowlesi* has made it possible to validate *P. vivax* vaccine candidates by replacing *P. knowlesi* genes with their *P. vivax* counterparts (15). In addition, HEK293E mammalian cell expression was applied for correctly folded recombinant *P. vivax* proteins with post-translational modifications resembling native parasites (16, 17).

High-throughput analysis of *P. vivax* ectodomain libraries previously identified fundamental molecular interactions, including Pv12-Pv41 and novel Pv12-PVX_110945 (hypothetical protein) (8, 17), which were predicted to have essential roles in blood-stage parasites. Pv12 belongs to the 6-Cys gene family, which includes proteins released onto the surface of merozoites tethered by glycosylphosphatidylinositol (GPI)-anchor and forming interactions with merozoite membrane-bound proteins such as Pv41 (17, 18). Pv12 and Pv41 responded strongly to *P. vivax-*infected Korean patients’ sera with 49% and 62,5% seropositivity, respectively (18, 19). Thus, the predicted complex formation involving Pv12 (rhoptry), Pv41 (surface), and a hypothetical protein, PVX_110945 (microneme in this study) is expected to be sequentially released during erythrocyte invasion and elicit a high immune response to vivax patients.

This study is the first to characterize PvMP38 (PVX_110945, estimated 38 kDa) as a novel micronemal protein involved in a complex formation with Pv41 and Pv12. It plays an essential role in understanding the parasite’s invasion mechanism with its subcellular distribution. To further explore this, *P. knowlesi mp38,* a homolog of *Pvmp38*, was knocked out via CRISPR/Cas9 and systematically tested the invasion inhibition activity of different antibody cocktails, allowing us to precisely attribute effects to direct targeting of MP38.

## RESULTS

### Full-length ectodomain protein expression and polyclonal antibody production

**Fig 1:**
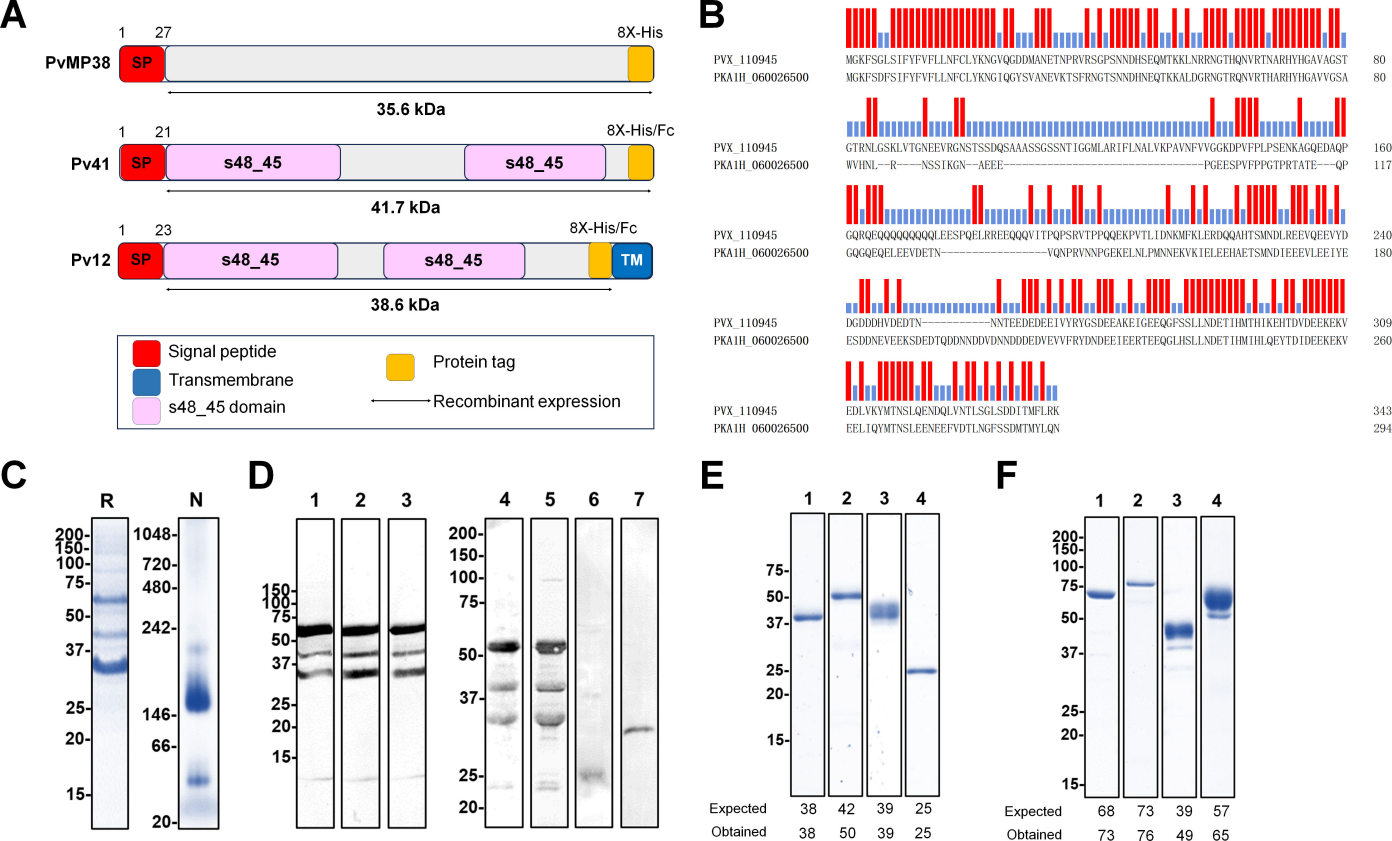
Recombinant protein expression. (A) Schematic structure of target proteins. (B) Amino acid sequence alignment of PVX_110945 (PvMP38) with orthologs in *P. knowlesi*, PkA1H_060026500 (PkMP38), the similarity is highlighted in red. The percent identity and divergence were analyzed using Clustal-W. (C) Recombinant PvMP38 (~37.8 kDa with 8× His tag) under reducing (R) and native condition (N). (D) Western blot analysis of recombinant PvMP38 probed with anti-His tag antibody for the positive control (lane 1), mouse- (lane 2), and rabbit-immune sera (lane 3). Recognition of the native PvMP38 antigen in the *P. knowlesi* schizont parasite lysate with mouse and rabbit antisera raised against the recombinant PvMP38 under reducing conditions (lanes 4 and 5) and the specificity confirmation by pre-immune mouse and rabbit sera (lanes 6 and 7). For protein-protein interaction analysis, other proteins were expressed with His-tag (E) and Fc-tag (F). Pv12 and Pv41 were expressed under His-tag (E, lanes 1 and 2) and were fused with Fc-tag (F, lanes 1 and 2). For positive and negative controls of protein-protein interaction analysis, PvDBP-RII-His (E, lane 3), human DARC-Fc (F, lane 3), and GST under His- or Fc-tags (E and F, lanes 4) were also expressed, respectively.

As proof of concept for this study, which targets protein function and establishes PPI systems for *P. vivax,* the full-length extracellular domains of recombinant proteins were fused with His- or Fc-tags following the schematics shown in Fig. 1A. The phylogenetic relationship exhibited a pairwise identity of 55.5% with our research model, *P. knowlesi* (Fig. 1B) and had a high degree of conservation among its orthologs in other *Plasmodium* species (Fig. S1). The expressed recombinant His-tagged PvMP38 protein was evaluated for denatured by SDS-PAGE and native antigens by BN-PAGE (Fig. 1C). Recombinant PvMP38 protein was consistently detected as three bands, approximately 35, 43, and 63 kDa, under denaturing conditions. These three bands were confirmed using LC-MS analysis and matched the sequence of the identified protein ID, XP_001608414.1, with extensive homology (*P*-value *<* 0.05) (Table S1). The native gel analysis showed PvMP38 migrated at a larger-than-expected size of approximately 150 kDa, while the predicted monomer molecular weight was 38 kDa, indicating oligomerization. Animal immune sera against PvMP38 were immunoblotted using recombinant protein and *P. knowlesi* schizont-enriched lysates for the specificity of produced antisera (Fig. 1D).

The expressed His- or human IgG1-Fc-tagged ectodomains of Pv12 and Pv41 were analyzed under reducing conditions (Fig. 1E and F, lanes 1 and 2). Under native conditions, their His-tagged forms appeared as major band sizes over 66 kDa, suggesting homodimer formation (Fig. S2). This has previously been observed in *P. vivax* P12 and P41 (17). As a positive control for the sensitivity of PPI systems (biolayer interferometry [BLI] and enzyme-linked immunosorbent assay [ELISA]), PvDBPII-His (194–521 aa) and human DARC-Fc (1–63 aa) were expressed (Fig. 1E and F, lanes 3). Two GST proteins tagged with His and Fc fragments, respectively, were used as a negative control for interaction specificity (Fig. 1E and F, lanes 4).

### *In vitro* PPI identification of PvMP38-Pv12 and/or Pv41

**Fig 2:**
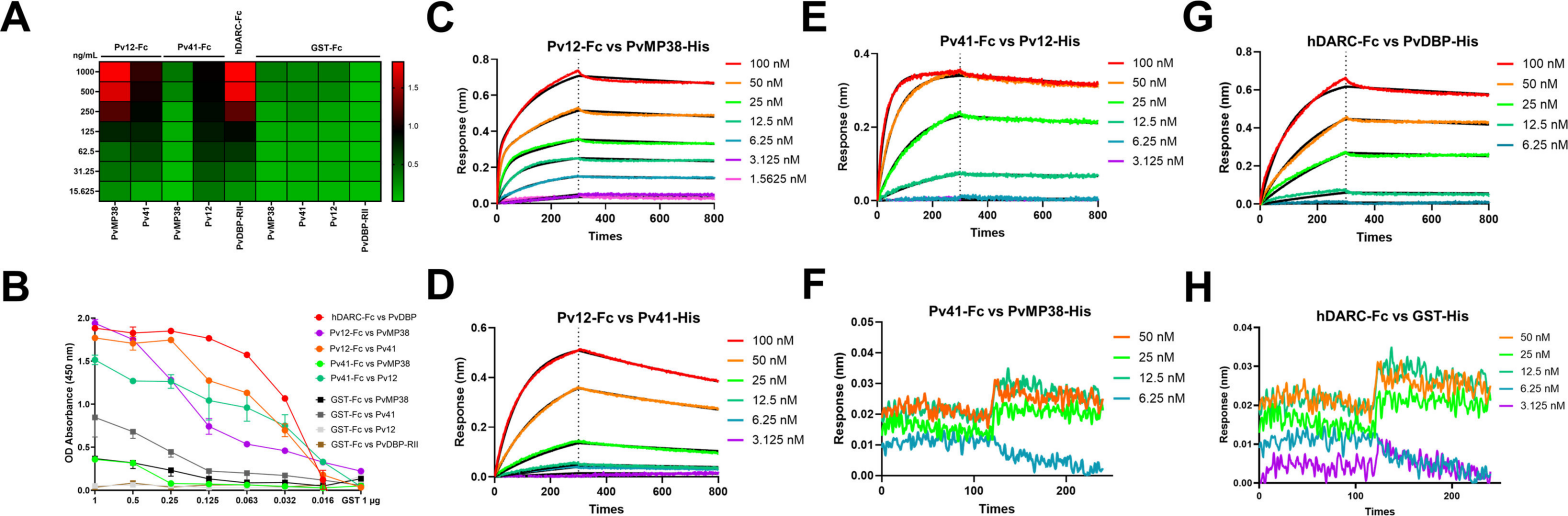
Identification and quantification of the protein-protein interactions. (A) Analysis of the interaction between Fc- and His-tagged recombinant protein by ELISA. (B) A line graph represents the ELISA absorbance of panel A; data points represent the means of two technical replicates, and error bars represent SD. (C–H) The recombinant His-tagged protein of each testing pair was injected over immobilized Fc-tagged recombinant protein. Raw data from two technical replicates of BLI experiments with different batches of protein in Table S2.

To measure PPI, ELISA and BLI experiments were performed (Fig. 2; Fig. S3; Table S2). ELISA detected the specific interactions between *P. vivax* P12 to Pv41 in both orientations and Pv12-Fc with PvMP38-His tag, but not Pv41-PvMP38 (Fig. 2A and B). In biochemical analyses of PPI, BLI showed a strong binding between PvMP38 and Pv12-Fc with a *K*_*D*_ value of 2.46 ± 0.12 nM but no detectable interaction with Pv41-Fc (Fig. 2C and F). The binding affinities of Pv12-Fc for Pv41 were measured at a *K*_*D*_ value of 14.15 ± 2.76 nM (Fig. 2D), while the reverse orientation resulted in a significantly higher affinity (*K*_*D*_ = 0.53 ± 0.03 nM) (Fig. 2E). This BLI data indicated that although Pv12 interacted with both Pv41 and PvMP38, Pv41 did not bind PvMP38, with the equilibrium binding experiments between *P. vivax* P12 is showing a clear trend of dissociation saturation, suggesting the interaction’s specificity. The positive interaction between DARC-Fc and PvDBPII-His was detectable at *K*_*D*_ = 2.70 ± 0.43 nM without non-specific binding with GST-His protein (Fig. 2G and H). Interspecies binding compatibility was demonstrated as *P. knowlesi* P12 interacted with PvMP38 and Pv41 by recapitulating interactions using recombinant Pk12 (Fig. S3A through C). Although the native proteins had greater masses, no self-binding or non-specific interaction is observed with the negative control, GST protein (Fig. S3D).

### Micronemal subcellular localization of PvMP38 in *P. vivax* and *P. knowlesi*-infected erythrocytes

**Fig 3:**
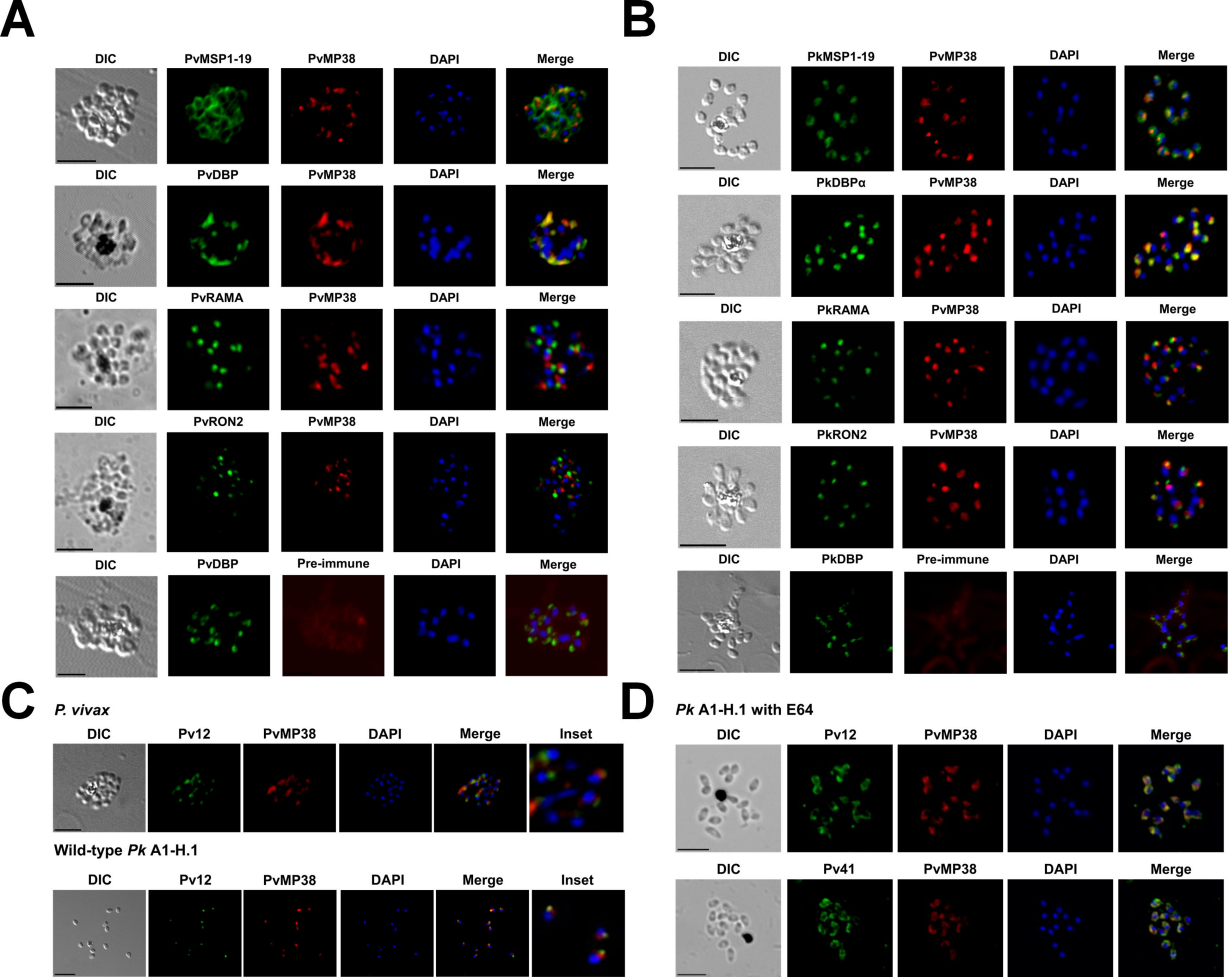
Subcellular localization of anti-PvMP38 with organelle-specific antibodies in the schizont stage of *P. vivax* (A)- and *P. knowlesi* (B)-infected parasites by immunofluorescence assay. (A) Reactivity of PvMP38 mouse polyclonal antibody (red) to *P. vivax* schizont-stage parasites co-localized with *P. vivax* surface protein, PvMSP1-19, microneme protein, PvDBP-II, rhoptry body (PvRAMA) and rhoptry neck protein (PvRON2) (each in green). (B) Cross-reactivity of mouse anti-PvMP38 (red) to *P. knowlesi* A1-H.1 schizont-stage parasites as red color co-localized with *P. knowlesi* surface protein, PkMSP1-19, microneme protein, PkDBPα-II, rhoptry body (PkRAMA) and rhoptry neck protein (PkRON2) (each in green). All samples were counterstained with antibodies raised from rabbits. (C) *P. vivax* and *P. knowlesi*, and (D) E64-treated *P. knowlesi* parasites were dual-labeled with rabbit antisera against Pv12/Pv41 (green) and mouse anti-PvMP38 (red). “Inset” panels depict enlarged regions. Non-specific staining is detected using PvMP38 pre-immune serum, proving the specificity of the produced antibody. Bars represent 5 mm. DAPI, 4',6'-diamidino-2-phenylindole.

Antibodies raised against the recombinant PvMP38 were used to explore the localization of MP38 within parasites. The anti-PvMP38 antibody specifically recognized *P. vivax* (Fig. 3A) and *P. knowlesi* parasites (Fig. 3B). These results showed that PvMP38 was co-localized with micronemal DBP (Fig. S4), displaying a distinct signal to rhoptry body RAMA, rhoptry neck RON2, and surface MSP1. The cross-reactivity between antibodies against recombinant *P. vivax* and *P. knowlesi* MP38 proteins was observed, indicating similar subcellular localization of both parasites. In addition, subcellular localizations of PvMP38 and Pv12 were analyzed from *P. vivax* and *P. knowlesi* WT (Fig. 3C) and E-64-treated parasites (Fig. 3D). Pv12 and PvMP38 were found to be located in adjacent apical loci, consistent with their respective localizations in rhoptry (Pv12) and microneme (PvMP38). This was seen in both segmented schizonts and egressed parasites. E64 treatment blocks egress but does not block the secretion of apical organelles; therefore, after incubation with this inhibitor, both MP38 and Pv12 were observed to be secreted onto the merozoite surface, where they then co-localized.

### Impact of *pkmp38* deletion on blood-stage invasion efficiency and growth in *P. knowlesi*

**Fig 4:**
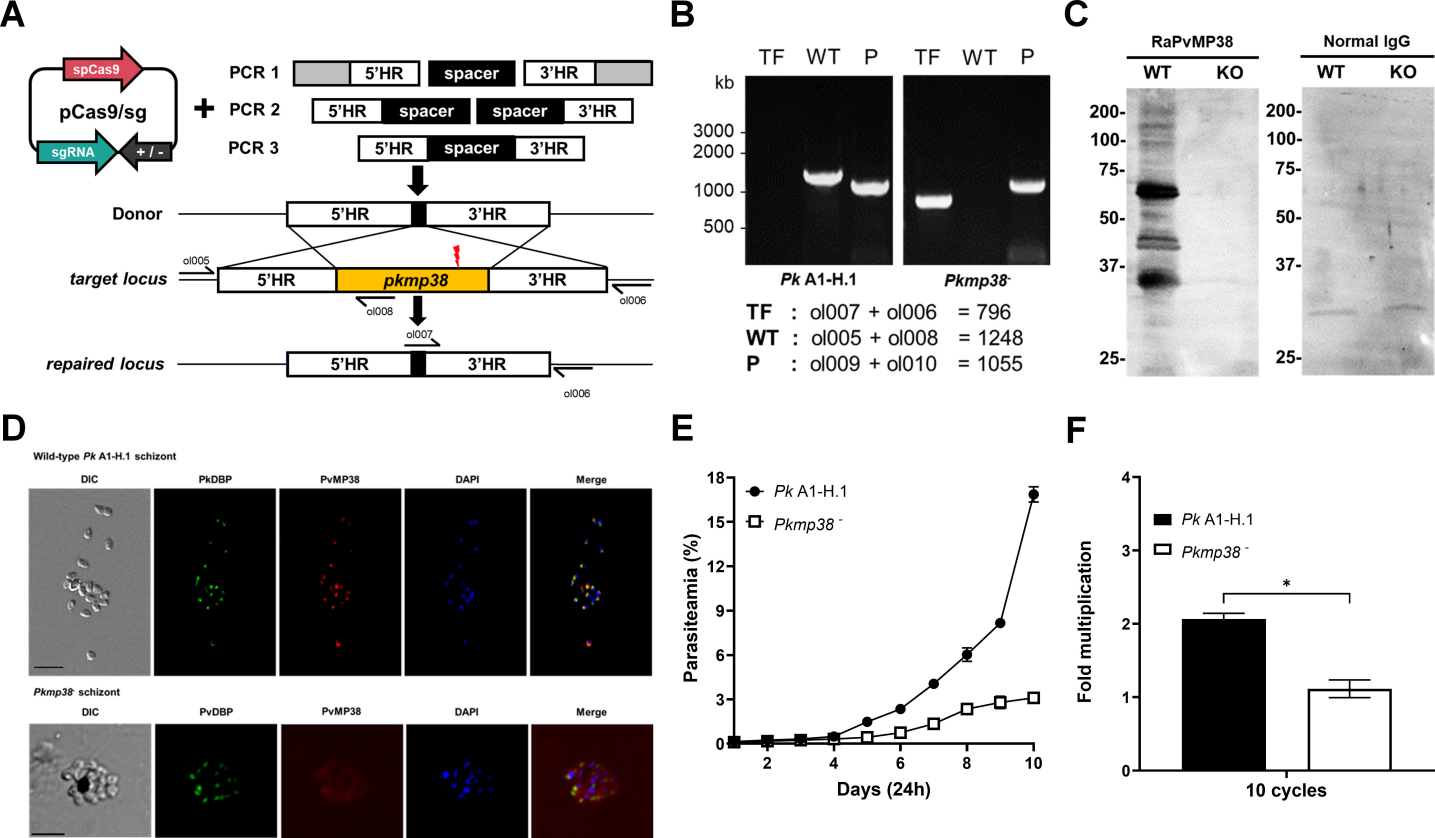
Disruption of *pkmp38*, an orthologue of *pvmp38,* in *P. knowlesi* parasites. (A) The donor DNA fragment was co-transfected with the pCas/sg construct that confers Cas9 nuclease, sgRNA, and drug resistance for further selection to knock out the *pkmp38* gene by double cross-over homologous recombination. The yellow box represents the barcode gene absent in the *P. knowlesi* genome for genotyping. Small black arrows indicate the primers. (B) Genotyping of parasite cloned out by limiting dilution. Target and disrupted fragments shown in panel A were amplified with primer pairs indicated in the box. TF, transfected parasites; WT, wild type; P, housekeeping gene. (C) Western blot analysis of WT and KO parasite lysates. Anti-PvMP38 recognized native antigens in *P. knowlesi* WT but not in KO parasite lysates. Non-immune rabbit IgG was used as a negative control. (D) Co-localization in *P. knowlesi* A1-H.1 (WT) and *Pkmp38^-^*(KO) using polyclonal antibodies against PvMP38. Rabbit anti-PkDBP antibody (green) was dual labeled with mouse anti-PvMP38 antibody as a localization marker. (E and F) Parasitemia and multiplication rate at 10 cycles of *P. knowlesi* WT and KO were measured. After recovery from the knockout process, the initial parasitemia was diluted to less than 0.1% and followed up for 10 days. Significant differences in the effects of pre-immune sera and other antibodies were calculated using an unpaired *t-*test*, ns*, not significantly different *P* > 0.05; *, *P* < 0.05.

To functionally characterize novel blood-stage antigens of *P. vivax*, we generated a CRISPR-Cas9-mediated knockout of *pkmp38*, the *pvmp38* ortholog*,* in *P. knowlesi*. The *pkmp38* gene was knocked out using a standard two-plasmid CRISPR-Cas9 system (Fig. 4A), and successful deletion was confirmed by genotyping with *pkmp38* locus-specific primers (Fig. 4B; Table S3). The WT *Pk* A1-H.1 parasite exhibited the expected band size containing the *pkmp38* gene, while the transfected parasite displayed the absence of this region, consistent with gene deletion verified by genome sequencing of WT and *Pkmp38^-^* (KO) (Fig. S5A). The absence of PvMP38 ortholog expression in KO strain was demonstrated by the lack of specific signal in KO parasites in immunofluorescence assay (IFA) and immunoblotting using anti-PvMP38 antibody, whereas WT parasites displayed clear signals (Fig. 4C and D; Fig. S5B and C).

Growth inhibition assays revealed a significant reduction in the growth rate and replication efficiency of KO parasites compared to WT during the initial 10 replication cycles (Fig. 4E and F; Fig. S5D); however, KO parasites were allowed to recover growth rate after approximately 15 replication cycles (Fig. S5E). These findings establish that *pkmp38* plays a critical role in blood-stage parasite growth and invasion. In addition, the ability of KO parasites to adapt suggests that *P. knowlesi* can employ alternative pathways for erythrocyte invasion in the absence of PvMP38 ortholog expression.

### PPI among Pv12, Pv41, and PvMP38 from *P. knowlesi* WT and KO parasites

**Fig 5:**
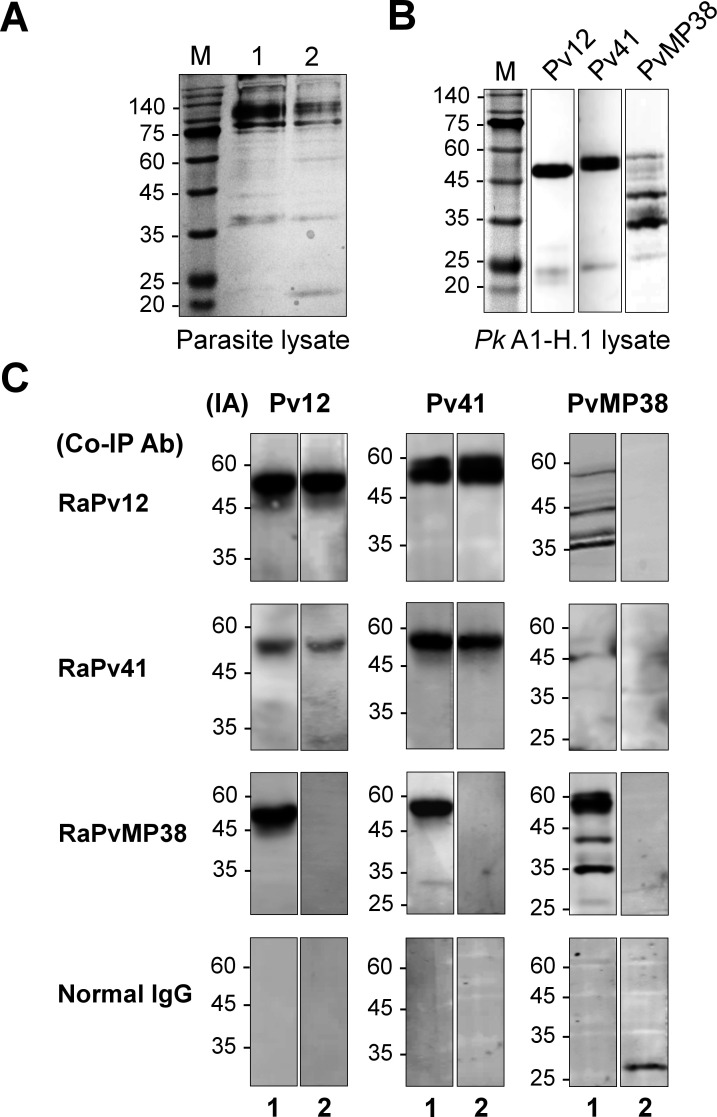
Co-immunoprecipitation assay using rabbit polyclonal antibodies (Co-IP Ab). (A) Total protein lysate from wild-type and knockout parasites were extracted and separated by 13% SDS-PAGE under reducing conditions, and followed by silver staining. (B) Specificity of mouse anti-Pv12, anti-Pv41, and anti-PvMP38 antibodies to recognize their orthologs in *P. knowlesi* by western blot analyses. (C) Western blot analyses were performed using mouse-raised antibodies to detect immunoprecipitated antigens (IA) in wild-type (lane 1) and knockout (lane 2) parasite, pulled down by rabbit sera against Pv12 (RaPv12), Pv41 (RaPv41), or PvMP38 (RaPvMP38). Normal rabbit IgG was used as a negative control for the co-immunoprecipitation assay.

Co-immunoprecipitation assays were conducted to validate the interactions among Pv12, Pv41, and PvMP38 using WT and KO schizont-rich *P. knowlesi* parasite lysates (Fig. 5A). Initially, the presence of orthologs for Pv12, Pv41, and PvMP38 was confirmed in WT parasites (Fig. 5B). Western blot demonstrated that rabbit anti-Pv12 and -PvMP38 antibodies successfully co-immunoprecipitated both Pv41 + PvMP38 and Pv12 + Pv41 orthologs in WT parasites, respectively. While using rabbit anti-Pv41 antibody could pull down Pv12 and Pv41, but not PvMP38 (Fig. 5C). This could be attributed to the low affinity of anti-Pv41 antibody for the complex or steric hindrance, which might reduce the accessibility of the antibody when all three proteins are present. These limit the ability of the anti-Pv41 antibody to precipitate the entire complex, reflecting in the lower intensity band in the Pv12 signal (Fig. 5C, RaPv41). In KO parasites, only Pv12 and Pv41 bands could be detected using anti-Pv12 and -Pv41 antibodies, respectively. These results provide evidence for complex formation among Pv12, Pv41, and PvMP38 and highlight the conserved interspecies-specific epitopes that are presented within these antigens.

### Immunoreactivity and epitope specificity of PvMP38 in *P. vivax* and *P. knowlesi*-infected patients

**Fig 6:**
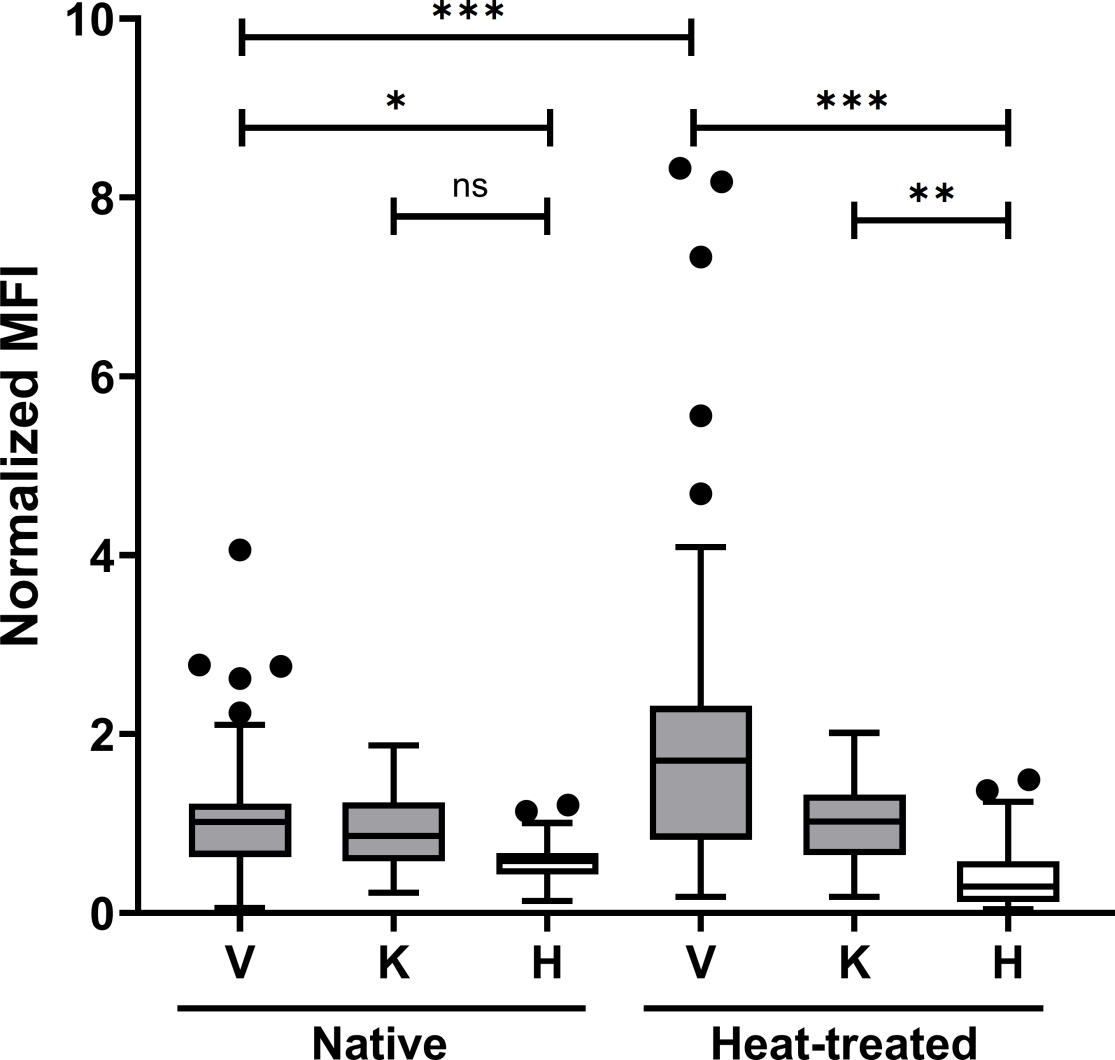
Humoral response of heat-treated and native PvMP38 to knowlesi (K) and vivax (V) patient sera. Normalized MFI represents the mean fluorescence intensity (MFI) divided by the cutoff value (equal to the mean fluorescence intensity plus two standard deviations (SDs) of the malaria-naïve samples). The bar indicates the mean ± standard deviation. Individuals with outlier reactivity were indicated in the black dot. The prevalence of antibody response was compared to the patients and healthy individuals (H) using the multi-variant one-way ANOVA and Tukey’s secondary test, ns, not significantly different *P* > 0.05; *P < 0.05; **P < 0.001; ***P < 0.0001.

To investigate the immune responses to PvMP38, a protein microarray was utilized to assess immunoreactivity against the full-length recombinant PvMP38 protein. Among *P. vivax* samples (*n* = 72), an IgG prevalence of 51.4% was observed (mean fluorescence intensity [MFI] =1.076 ± 1.370, 2 SD), while *P. knowlesi* samples (*n* = 56) exhibited an IgG response rate of 41.1% (MFI = 0.938 ± .834). Comparison between native and heat-treated antigens revealed that the immune response to PvMP38 predominantly targeted linearized epitopes. Denatured PvMP38 elicited a significant increase in immune responses from *P. vivax* patients, with seroreactivity rising from 51.4% to 70.8% (MFI = 2.001 ± 3.360), while a modest increase was observed for *P. knowlesi* samples, with sensitivity rising to 51.8% (MFI = 1.016 ± .838) (Fig. 6; Table S4). Thus, IgG responses to PvMP38 in *P. vivax* patient sera demonstrated high specificity compared to malaria-naïve sera, showing statistically significant differences. These findings highlight the immunogenicity of PvMP38 and its linear epitope specificity in *P. vivax* and *P. knowlesi* infections.

### Additive inhibition of *P. knowlesi* merozoite invasion by antibodies targeting PvMP38, Pv12, and Pv41

**Fig 7:**
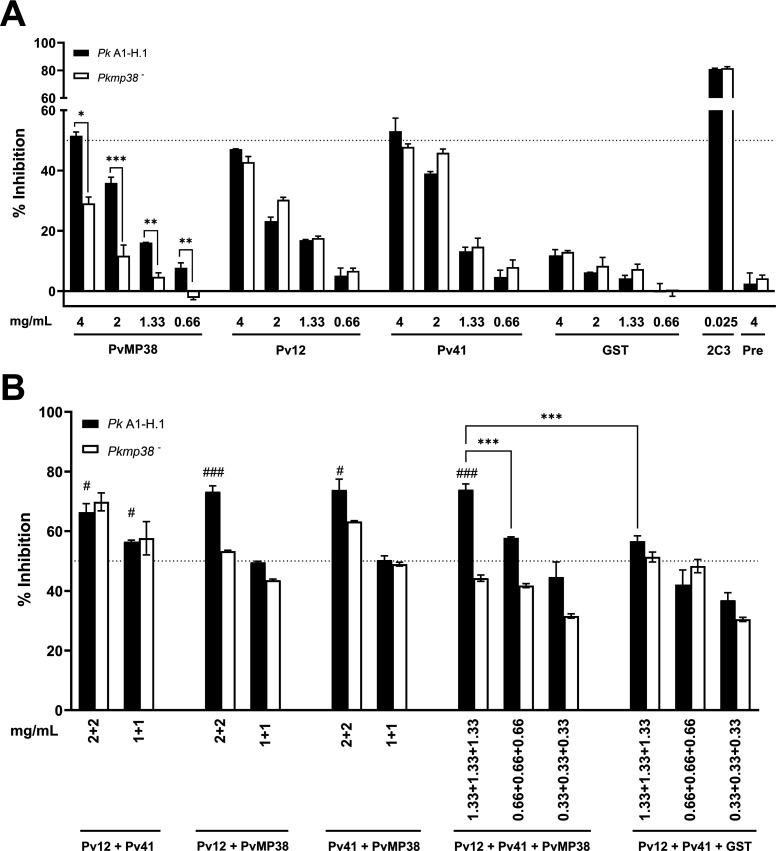
The invasion-inhibitory activity of *P. vivax* antibodies in single (A) and combination (B) against *P. knowlesi* A1-H.1. (A) Total IgGs purified from rabbit sera were tested individually for invasion-inhibitory activities (0.66 to 4.0 mg/mL). (B) Combinations of two IgGs were assessed at two concentrations (2.0 and 2.0 mg/mL, and 1.0 and 1.0 mg/mL), and combinations of three IgGs were tested at 0.33, 0.66, and 1.33 mg/mL each. 2C3 IgG (25 µg/mL) was used as a positive control. To determine whether the inhibition was statistically significant, all treatments were independently compared to the same concentration of total IgG of the negative control, which is the inhibition of purified IgG from pre-immune rabbit sera (single) combined with GST-his rabbit immunized (combination). Two independent assays were performed in duplicate. Significant differences in the effects of pre-immune sera and other antibodies were calculated using a multi-variant one-way ANOVA and Tukey’s secondary test, ^*/#^*P <* 0.05; **^/##^*P <* 0.01; ***^/###^*P <* 0.001. * represents the difference among groups within the graph. # represents the difference between single and combination antibody strategies, where 2 + 2 and 1 + 1 double combination sets (2 mg/mL and 1 mg/mL of each antibody) were compared with 4 mg/mL and 2 mg/mL of its single antibody components. A triple combination set of 1.33 + 1.33 + 1.33, 0.6 mg/mL, and 0.3 mg/mL each were compared with three single antibodies at 4, 2, and 1 mg/mL.

This study identified PPIs involving the conserved epitope shared by *P. vivax* P12, P41, and MP38 orthologs in *P. knowlesi* parasite. To explore potential additive effects, we assessed combined antibody responses against WT and KO *P. knowlesi parasites* using purified polyclonal antibodies, tested individually and in combinations (double antibody and triple antibody) (Fig. 7). A single PvMP38 antibody exhibited dose-dependent inhibition of merozoite invasion, reaching 51.55% ± 1.25% at 4 mg/mL in WT parasites. By contrast, the invasion inhibition in the KO parasite line was significantly reduced to 29.08% ± 2.15%, confirming that the PvMP38 antibody specifically targets its antigen (Fig. 7A). While the anti-Pv12 antibody alone showed less than 50% inhibition, its combination with other antibodies at 2 mg/mL significantly enhanced inhibition, with efficiencies ranging from 64.77% ± 0.05% to 65.76% ± 0.90% (Fig. 7B). Among the double antibody combination, the combination of anti-Pv41 and anti-PvMP38 antibodies (2 mg/mL each) inhibited invasion by 63.22% ± 2.01%, comparable to the anti-Pv12 plus anti-PvMP38 antibody combination. The most potent inhibition was observed with the triple-antibody combination (Pv12, Pv41, and PvMP38, at 1.3 mg/mL each), which resulted in 73.95% ± 1.91% inhibition, outperforming individual antibodies at 4 mg/mL in WT parasites, indicating an additive inhibitory effect. In KO parasites, the antibody cocktail containing anti-PvMP38 antibodies reduced inhibition efficiency, while the Pv12 + Pv41 combination remained effective, suggesting that the other members of the complex remained critical targets in the absence of PvMP38. Furthermore, antibody combinations with anti-GST polyclonal antibodies exhibited no significant inhibition, confirming that the observed additive effect is specific to the Pv12-Pv41-PvMP38 complex.

## DISCUSSION

Compared with *P. falciparum*, few *P. vivax* antigens have been described during the parasite invasion process due to the lack of a continuous *in vitro* culture system. Previous studies have investigated the molecular and cellular interaction of four important parasite ligands and erythrocyte (reticulocyte) receptors such as PvDBP-DARC (20), PvRBP2b-CD71 (21), PvRBP2a-CD98 (22), and PvEBP and CR1 (23)—making it clear that *P. vivax* invades host erythrocytes by establishing multiple distinct interactions with host receptors. Our previous reports identified important novel vivax molecules related to host cell invasion, including merozoite surface (19, 24), rhoptry (18, 25, 26), microneme (27, 28), dense granule, food vacuole (29), caveola-vesicle complexes (30), etc. However, many proteins, including PvMP38, remained poorly characterized.

In a previous study, a *hypothetical protein*, PvMP38, was briefly described; its transcription and genomic location suggested a function in merozoite development or erythrocyte invasion process; however, the extremely weak PPI with Pv12 neglected further study (17). In this study, we characterized PvMP38, a micronemal protein, by leveraging improved recombinant protein quality for PPI analysis and genetic modification in *P. knowlesi* parasites. To strengthen molecular interaction between these proteins, we employed a genetically engineered IgG Fc domain tag on the protein-binding partner, effectively enhancing the interaction avidity (31). This expression strategy increased interaction affinity (*K*_*D*_ value) between Pv12 and Pv41 sixfold compared to a previous report and enabled us to detect the Pv12/Pk12-PvMP38 interaction with high affinity. IgGs against PvMP38 were reacted on the microneme region in both *P. knowlesi* and *P. vivax* parasites, suggesting that the PPI complex may form among three main components of merozoite: PvMP38 (microneme in this study), Pv12 (rhoptry) (18), and Pv41 (surface) (19). Our co-immunoprecipitation assay further supported this hypothesis by demonstrating the mutual pull-down of these antigens, providing evidence for their interaction within the parasite, and IFA offered additional proof of the complex localization. Previous IFA studies of Pv12 in *P. vivax* (18) exhibited that it localizes to the rhoptry rather than the merozoite surface. Conversely, an experiment with *P. knowlesi* showed it resides in the merozoite surface (13). Given protein secretion during the invasion, the E64-treated parasite was used to observe the translocation of target antigens to the merozoite surface. Interestingly, both Pv12 and PvMP38 were secreted from the apical organelle onto the E64-treated parasite surface and overlapped with the Pv41 signal, supporting co-localization and the potential for these proteins to form a complex during invasion (Fig. 3C and D). However, attempts to confirm these PPIs through re-orientation using Fc-tagged PvMP38 were unsuccessful due to precipitation caused by the increased protein size. There is currently no evidence for the structural configuration of this protein complex. Thus, experiments aimed at tracking the co-localization of these interactions within the parasites and/or structural analysis by cryo-EM may provide valuable insights into the biological evidence of these interacting pairs.

LC-MS analysis suggested the presence of truncated forms or structural isoforms of PvMP38, as the protein displayed multiple molecular weights under reducing conditions and formed oligomers on native PAGE. These differences could result from disulfide bond patterns or variations of hydrodynamic structure, indicating either a natively folded conformation or an elongated structure. Further investigation was conducted to define whether PvMP38 contains conformational or linear epitopes through heat treatment and assessed its serological activity in *P. vivax* and *P. knowlesi-*infected patients. Heat treatment denatured and disrupted the PvMP38 structure, exposing hidden linear epitopes, which led to a 20% increase in IgG reactivity in the vivax-positive patient serum samples, indicating that PvMP38 targets linear epitopes. Such structural insights would aid in distinguishing protective versus conformational epitopes, which could significantly impact vaccine candidate identification. Further research on these targets will benefit from target-specific methods, such as monoclonal antibodies or peptide mapping. Larger sample size screenings and assessment of antigen-specific IgG response durations following the *P. vivax* exposure would be needed to determine the scope of the IgG response in providing clinical defense.

For the first time, it was confirmed that the PvMP38 forms conserved PPIs or protein complexes with Pv12 and Pv41 across species, evidenced by cross-reactive interactions with *P. knowlesi* orthologs. We cannot rule out that PvMP38/Pk12 and Pk12/Pv41 form mutually exclusive complexes; however, this interaction is also crucial for functional activity and likely predates the evolutionary divergence of *P. knowlesi* and *P. vivax*. The co-immunoprecipitation revealed the complex formation among Pv12, Pv41, and PvMP38 in *P. knowlesi* using antibodies against *P. vivax* antigen, implying the existence of conserved interspecies epitopes within these antigens. The high conservation led us to use *P. knowlesi*, a human erythrocyte-adapted alternative model for *P. vivax.* Previous reports have emphasized the suitability of *P. knowlesi* as a model for *P. vivax* vaccine development (12, 13, 15, 31–34). This study successfully utilized the strengths of *P. knowlesi* to assess antibody efficacy and genetic tractability without a robust *P. vivax in vitro* cultivation system. However, some *P. vivax* proteins have orthologs in *P. knowlesi* that exhibit strong cross-reactivity; others do not, and certain *P. knowlesi* genes are not amenable to knockout, complicating research efforts (13). Therefore, employing various models, including the use of ortholog replacement approaches on *P. knowlesi*, *P. cynomolgi*, and *P. vivax ex vivo*, as well as transgenic rodent models, could provide a more precise approach to characterizing *P. vivax* antigens.

Gene KO experiments in *P. falciparum* P12 and *P. knowlesi* P41 have shown that these genes are not essential for invasion (13, 35). However, the inability to delete *pk12* in *P. knowlesi* in the previous study suggests that the *pk12* gene is essential for invasion and development (13). Thus, the functional role of Pv12 alternatively could mainly be evaluated through invasion inhibition assay (IIA) by target-specific IgG. In this study, *pkmp38* gene deletion hampered invasion and growth initially, but it was compensated by alternative invasion molecules in *P. knowlesi*. The slow recovery after the target gene KO process allows parasites to compensate for the loss of a specific gene. By contrast, IIAs validated in a single cycle may limit parasites from finding alternative ways to adapt. Understanding precisely how parasites are able to adapt to this pathway loss may also enable us to find synergistic combinations to block adaptation. Given that vaccines targeting a single candidate (e.g., PvDBP and PvMSP1-19) (36, 37) are vulnerable to immune system evasion, a systematic approach using a combination of antibodies was applied to enhance protection against malaria. The additive inhibition effect of triple antibodies against PvMP38, Pv12, and Pv41 suggests a function in targeting distinct epitopes.

This study highlights the discovery of the novel micronemal protein PvMP38 and its conserved interactions with Pv12 and Pv41, alongside advancements in Fc-fusion protein expression and enhancing binding avidity in PPIs. Notably, the identification of PvMP38 represents a significant breakthrough, providing deeper insights into the mechanisms of *P. vivax* reticulocyte invasion. PvMP38 offers a new avenue for vaccine development, supporting a multi-antigen approach to improving immune responses and protection against malaria.

## MATERIALS AND METHODS

### Collection of malaria-infected patients’ serum samples

*P. vivax* patient samples from the Republic of Korea from 2009 to 2011 and *P. knowlesi* patient samples from *P. knowlesi* endemic areas at the University of Malaya Medical Center (UMMC), Malaysia, between 2010 and 2013 were collected for humoral immune response analysis by protein array. A positive *P. vivax* sample from Thailand was used to purify parasites in the IFA. Healthy sera were from 10-year-old children with no malaria infection history from non-endemic regions in Gangwon Province Hospitals as a negative control. Information on parasite count, age, and sex obtained in this study is provided in Table S5.

### Design and gene synthesis for plasmid DNA construction

All gene sequence data and protein information were obtained from the Plasmodium Genomics Resource (PlasmoDB: http://plasmodb.org/plasmo/) and GenBank websites (Table S6). The expected molecular weights of the *P. vivax* proteins and orthologous proteins in *P. knowlesi* were predicted using SnapGene software (GSL Biotech LLC, San Diego, CA). The amino acid sequence data were aligned using the CLUSTAL-W program in MegAlign Lasergene software (version 7.0, DNASTAR, Madison, WI). Sequences encoding the codon-optimized full-length ectodomain were obtained from the Addgene library (Addgene Inc., Watertown, MA). The optimized sequences were cloned into the pTT5-based expression vector. For the His-tagged expression, the endogenous signal peptide (leader sequence of human variable light chain) in the upstream region combined with 8×His C-terminal tag. The Fc-fusion vector was generated by fusing the human immunoglobulin Fc domain (HIgGk-Fc) tag.

### HEK293 EBNA1-6E cell cultivation and recombinant protein purification

The HEK293-EBNA1 human cell line was adapted for suspension culture in FreeStyle F17 Medium (Life Technologies, Burlington, ON) at 37°C, 75% humidity, and 8% CO_2_ with shaking at 125 rpm. Expression plasmids were transiently transfected using PEI (Polysciences, Inc., Warrington, PA) (38, 39). For His-tagged protein purification, the culture supernatant was incubated with Ni-NTA resin (QIAGEN, Hilden, Germany) at 4°C overnight. The mixture was loaded onto a Poly-Prep Chromatography column (Bio-Rad Inc., Hercules, CA) and washed with 10 mM imidazole, 300 mM NaCl, and 50 mM sodium phosphate (pH 7.4), followed by elution with 250 mM imidazole. For Fc-tagged proteins, the supernatant was passed through a 1 mL HiTrap Protein G HP column (GE Healthcare, Uppsala, Sweden) and purified according to the manufacturer’s instructions. Elutates were buffer-exchanged into PBS using a 10 kDa Amicon Ultra-15 Centrifugal Filter (Merck Millipore, Darmstadt, Germany).

### Production of animal immune sera and antibody purification

Six-week-old female BALB/c mice and Japanese white rabbits were used to generate polyclonal antibodies. Mice were injected with 30 µg, and rabbits 250 µg of His-tagged recombinant protein intraperitoneally with Freund’s complete adjuvant (Sigma-Aldrich, St. Louis, MO), followed by Freund’s incomplete adjuvant (Sigma-Aldrich) at 3-week intervals. Immune rabbit IgGs were purified using a protein G HP column following the manufacturer’s protocol (GE Healthcare). The eluted IgG fractions were dialyzed into RPMI-1640 (Invitrogen/Gibco, Grand Island, NY) or PBS using a 10 kDa cutoff filter (Merck Millipore). Purified IgGs were pre-adsorbed with human O^+^ erythrocytes (25 µL of RBCs per 4 mg of IgGs) to remove non-specific inhibitory effects (32).

### Gel electrophoresis and western blotting analyses

For quality assessment of the purified proteins and parasite lysates, blue native (BN)/13% SDS-PAGE/silver stain and western blot analyses were performed (40, 41). Samples were boiled with reducing buffer for SDS-PAGE or prepared with sample buffer for 4%–16% native-PAGE (Thermo Fisher Scientific, Cleveland, OH) and stained with Coomassie brilliant blue (Sigma-Aldrich) or Pierce Silver Stain Kit (Thermo Fisher Scientific). Western blot was analyzed by incubating with primary antibodies: mouse anti-6×His tag (1:2,000, ab18184, Abcam, Cambridge, MA), mouse anti-PvMP38 (1:500), and rabbit anti-PvMP38 (1:1,000). The membranes were then detected by secondary antibodies including IR Dye 680RD goat anti-mouse IgG, IR Dye 800CW goat anti-rabbit IgG, (LI-COR Inc., Lincoln, NE,) and analyzed using the Odyssey infrared imaging system (LI-COR Inc.). For protein identification, bands were digested with trypsin, and peptides were analyzed by LC-MS/MS. Data were processed in Proteome Discoverer (v1.4) using Mascot v2.4 (Matrix Science, London, UK) with a cutoff ion score of 30 and a significance threshold of 0.05.

### Biolayer interferometry analysis

BLI was used to measure protein-protein binding affinity and kinetics using the real-time Gator Label-Free Bioanalysis System (Gator Bio, Palo Alto, CA) with anti-human IgG Fc (HFC) probes. The biophysical binding affinity of the predicted interactions was analyzed with the one-to-one fit model. The probes were activated, followed by baseline (PBS, 90 s), Fc-tagged protein immobilization (10 µM, 120 s), washing (PBS, 90 s), serial dilution of His-tagged protein (1.5 to 100 µM, 300 s), and dissociation (PBS, 600 s). Binding affinities (*K*_*D*_ values) were calculated using a 1:1 global fitting model, with glutathione S-transferase (GST)-His as a negative control to assess nonspecific binding.

### Enzyme-linked immunosorbent assay

For PPI analysis, Fc-tagged proteins (1 µg/mL) in a 0.1 M sodium carbonate buffer (pH 9.6) were coated to 96-well plates and incubated overnight. The interaction was assessed using serial dilutions of the partner proteins, and the interaction of hDARC-PvDBP and GST protein was used as positive and negative controls, respectively. The reaction detection was performed with HRP-conjugated anti-His tag antibodies (1:5,000), added substrate, and stopping the reaction; absorbance was measured at OD_450_ by a PHERAstar plus instrument (BMG Labtech, Germany).

### Serological analysis by protein microarray

As described previously, a protein microarray was performed to evaluate the antigenicity of PvMP38 in malaria patients and healthy individuals (42). Recombinant PvMP38 protein was applied onto the aminopropyl-coated slides at 100 ng/µL. The amine chips were probed with serum samples from *P. vivax-*infected patients (Republic of Korea, *n* = 72), *P. knowlesi-*infected patients (Malaysia, *n* = 56), and 70 healthy individuals, diluted 1:25 in PBS-T in duplicate. Finally, 10 ng/µL of Alexa Fluor 546-conjugated goat anti-human IgG (Invitrogen) in PBS-T was applied for data visualization. Fluorescent signals were scanned using InnoScan 300 (INNOPSYS, France). To identify the epitope of an antigen, recombinant PvMP38 was heat-treated at 100°C for 5 minutes and proceeded as described above.

### *In vitro* cultivation of *P. knowlesi*, synchronization, and native antigen extraction

The *P. knowlesi* human-adapted strain (PkA1H1_HB) strain was cultured in 2% hematocrit following the previous protocol (43) with human O-type erythrocytes in a complete RPMI-1640 medium with supplements and gentamicin (Invitrogen) (44). Mature schizont-stage parasites were enriched using a gradient (40%/70%) Cytiva Percoll solution (Thermo Fisher) (26). The schizont-rich parasite was lysed by saponin, as previously described (45). The extracted mixtures were fractionated by SDS-PAGE and blotted onto the PVDF membrane, which was probed with PvMP38 rabbit IgG at 1 µg/mL as the primary antibodies.

### CRISPR-Cas9 genome editing, transfection, and genotyping of *P. knowlesi* parasites

The templates were synthesized following the previous report (46). Briefly, the 5′ and 3′ homology regions (800 bp each) flanking the *pkmp38* guide were amplified with a 25-nucleotide overhang at the 3′ and 5′ ends from *P. knowlesi* genomic DNA. The final templates were pooled, ethanol-precipitated, and used as the 5′ HR-*pkmp38*-3′ HR construct for transfection. Three single-guide RNAs (sgRNAs) were screened using CRISPR REGEN Tools (47) and selected with high out-of-frame scores and a single on-target effect (Table S7). The pCas9/sg (15) vector was produced by cloning the annealing sgRNAs into BtgZI digested Cas9 vector. Primer sequences, primer combinations, and running conditions are listed in Tables S3, S8, and S9.

The tightly synchronized *P. knowlesi* parasites schizont was transfected using the Amaxa 4D electroporator (Lonza, Basel, Switzerland) and the P3 Primary cell 4D Nucleofector X kit L (Lonza), as previously described (44). Briefly, 20 µg of synthesized template (PCR products) and 20 µg pCas9/sg were mixed with P3 Primary cell nucleofector solution, including supplement 1 (Lonza). The DNA with 10 µL packed parasites mixture was transferred to a Nucleocuvette Vessel (Lonza), followed by a nucleofection program, FP158. Transfected parasites were selected by drug treatment with 100 nM pyrimethamine (Sigma-Aldrich). The transfected parasites were cloned out by limiting dilution and culture until the parasite appeared.

### Indirect IFA analysis

The IFA was performed as described previously (12). Primary antibodies included mouse anti-PvMP38 (1:50) mixed with rabbit polyclonal antibodies specific to cellular locations of *P. knowlesi* and *P. vivax*: rabbit anti-RAMA (rhoptry body-specific antibody, 1:100), anti-RON2 (rhoptry neck-specific antibody, 1:100), anti-DBP (microneme-specific antibody, 1:250), and anti-MSP1-19 kDa (merozoite surface-specific antibody, 1:100). Secondary antibodies of Alexa 568-conjugated goat anti-mouse IgG and Alexa 488-conjugated goat anti-rabbit IgG (Invitrogen) were used at 1:500 dilution. Nuclei were stained with DAPI (4′,6′-diamidino-2-phenylindole, Invitrogen). *P. knowlesi* A1-H.1 schizonts were treated with 10 µM of cysteine proteinase inhibitor (E64) (Sigma-Aldrich) for 2 hours to prevent egress and tested protein secretion with anti-Pv12 antibody (1:100). Slides were visualized using a motorized inverted fluorescence confocal microscope *ECLIPSE Ti2* (Nikon, Tokyo, Japan). Each fluorescence graphic was calculated using Image J (NIH, Bethesda, MD).

### Invasion inhibition assay

*P. knowlesi* wild-type (PkA1-H.1, WT) and knockout (*Pkmp38*^−^, KO) strain schizonts, enriched by magnetic separation (MACS, Miltenyi Biotec, Rhine-Westphalia, Germany) were adjusted to 1%–2% parasitemia (48). Well without and with an anti-Fy6 monoclonal antibody against the Duffy antigen/receptor for chemokines (DARC; clone 2C3; 25 µg/mL; Absolute antibody, Wilton, UK) were defined as normal invasion and antibody-mediated invasion inhibition. After one cycle of parasite growth (approximately 10 hours), the pellets were fixed with 0.05% glutaraldehyde (Sigma-Aldrich) and then stained with 0.2× SYBR Green I (Sigma-Aldrich). Two hundred thousand events were analyzed using an Accuri C6 flow cytometer (BD Biosciences, San Jose, CA). Inhibition of invasion of tested wells was compared to purified pre-immune IgG and rabbit IgG against GST-His proteins with the same adjuvant. Two independent experiments were performed in duplicate.

### Co-immunoprecipitation

Co-immunoprecipitation was carried out using a Dynabeads Protein G Immunoprecipitation Kit (Thermo Fisher Scientific). Briefly, the Dynabeads-antibody complex was prepared by incubating rabbit polyclonal antibodies against Pv12, Pv41, and PvMP38 (diluted 1/100) with Dynabead Protein G for 30 minutes, rotation at RT; and normal polyclonal antibody was used for negative control. Then, the Dynabeads-Ab-antigen complex was prepared by adding the freshly extracted mature schizont of WT and KO parasites for 2 hours of rotation at RT. After washing twice with a washing buffer, proteins were eluted with an elution buffer and neutralized with 1 M Tris, pH 9.0. The supernatants were analyzed by western blot using mouse antibodies against Pv41, PvMP38, and Pv12 antibodies (diluted 1:500) as primary antibodies.

### Data analysis

Data are presented as mean plus standard deviation (SD) for duplicate technical and biological replicate measurements. The data were analyzed using GraphPad Prism software version 10.1.2 (GraphPad, San Diego, CA). Student’s *t*-test, a multi-variant one-way ANOVA, and Tukey’s secondary tests were used to compare experimentally measured statistical differences (*P*-values) among groups. All kinetic assay data were observed using Gator^®^ GatorOne v2.10 software (Gator Bio).

## SUPPLEMENTAL MATERIAL

Supplemental figures mbio.03917-24-s0001.pdf Fig. S1 to S5.

Supplemental tables mbio.03917-24-s0002.pdf Tables S1 to S9.

ASM does not own the copyrights to Supplemental Material that may be linked to, or accessed through, an article. The authors have granted ASM a non-exclusive, world-wide license to publish the Supplemental Material files. Please contact the corresponding author directly for reuse.
