# Supplementary material for: A novel micronemal protein MP38 is involved in the invasion of merozoites into erythrocytes
Source: mBio. 2025 Apr 9;16(5):e03917-24. doi: 10.1128/mbio.03917-24 (PMC12077092; doi:10.1128/mbio.03917-24)
Supplement: Supplemental figures — Fig. S1 to S5. [file mbio.03917-24-s0001.pdf]

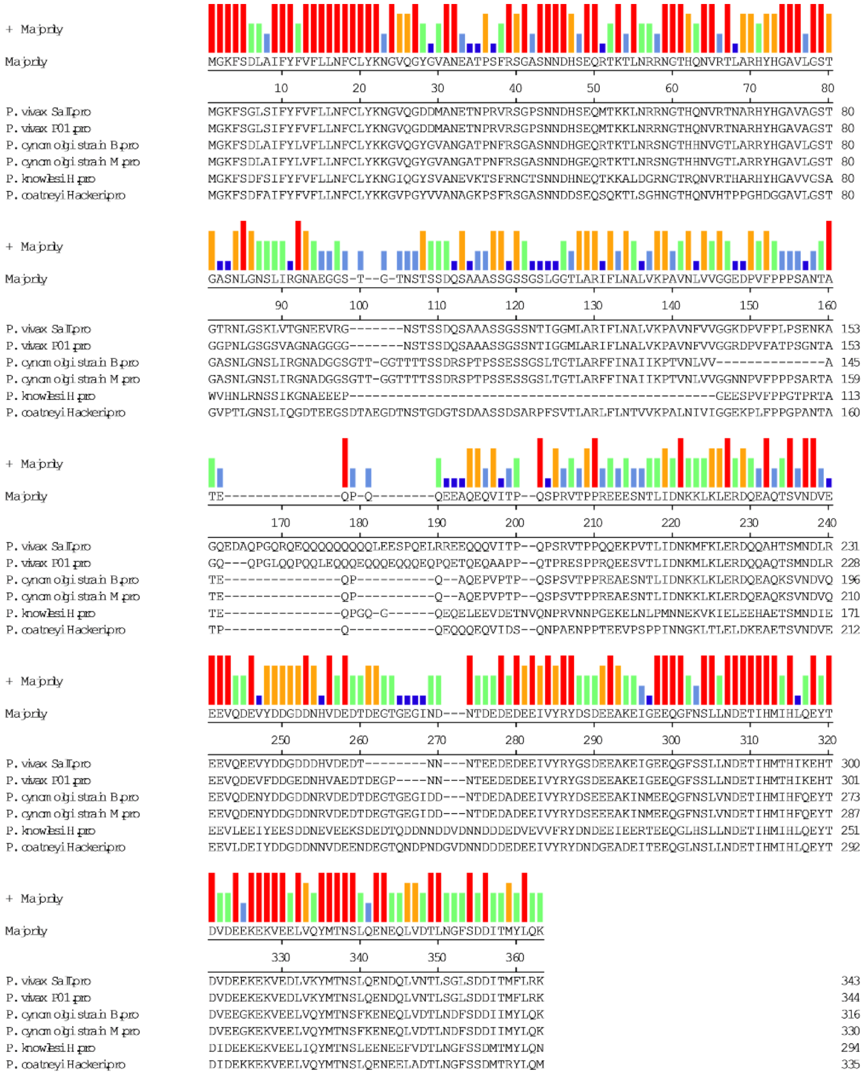

Percent Identity

Divergence	1	2	3	4	5	6		
1		86.5	63.9	63.6	54.1	56.8	1	*P. vivax* SalI
2	15		65.4	65.0	53.7	57.0	2	*P. vivax* P01
3	48.9	46.3		100.0	58.5	61.9	3	*P. cynomolgi* strain B
4	49.5	46.9	0.0		58.5	61.7	4	*P. cynomolgi* strain M
5	69.6	70.5	59.6	59.8		67.0	5	*P. knowlesi* H
6	63.4	62.9	52.8	53.2	43.3		6	*P. coatneyi* Hackeri
	1	2	3	4	5	6		

FIG S1. (A) Amino acid sequence alignment of PVX_110945 (PvMP38) in *P. vivax* SalI with orthologues in *P. vivax* P01, *P. cynomolgi* strain B and M, *P. knowlesi* strain H and *P. coatneyi* Hackeri, the similarity is highlighted in red. (B) The percent identity and divergence were analyzed using Clustal-W.

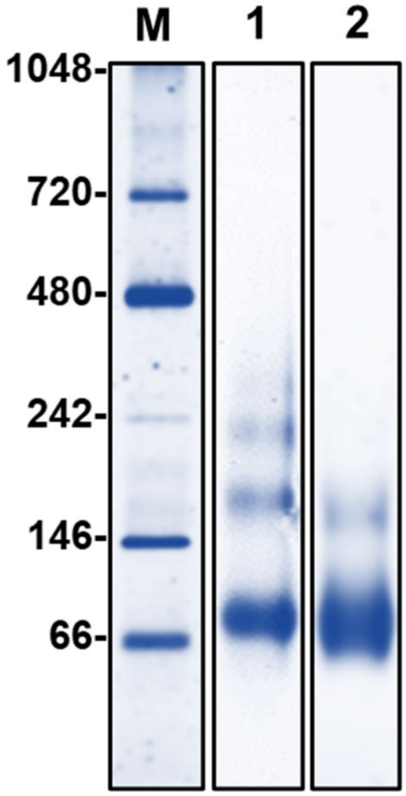

FIG S2. Pv12 and Pv41 exist as homodimers or oligomers. Recombinant His-tagged Pv12 and Pv41 (lanes 1 and 2) were run with BN-PAGE. The monomers of Pv12 and Pv41 His-tagged protein in denature condition are shown in Fig 1, support the presence of homodimers.

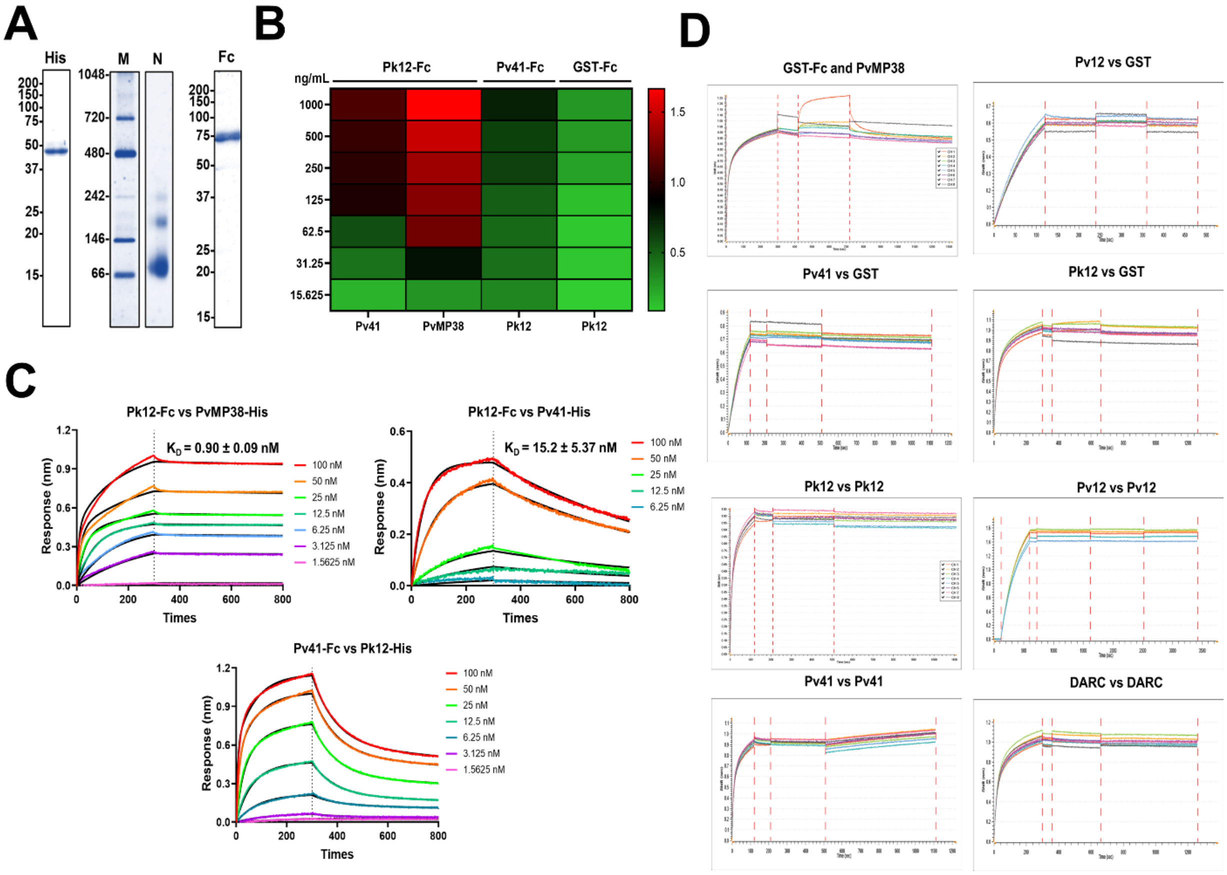

FIG S3. Interspecies binding compatibility between *P. knowlesi* P12 and PvMP38 or Pv41 was demonstrated by recapitulating the same interactions using recombinant Pk12. (A) Pk12 recombinant protein expression under His-tag (reducing and native (N) condition) and Fc-tag. The protein-protein interaction using ELISA (B) and BLI (C). (D) The specificity of the protein-protein interaction assay was measured by showing the binding with negative control, which are GST-Fc and PvMP38-His or between Fc protein and GST-His tagged. The same concentrations were used to check the self-interaction of Fc- and His-tagged forms itself. Although the native form of proteins has greater masses of the expected sizes, no self-binding or nonspecific interactions with GST protein are observed, proving that those proteins cannot self-form into the higher-order structure.

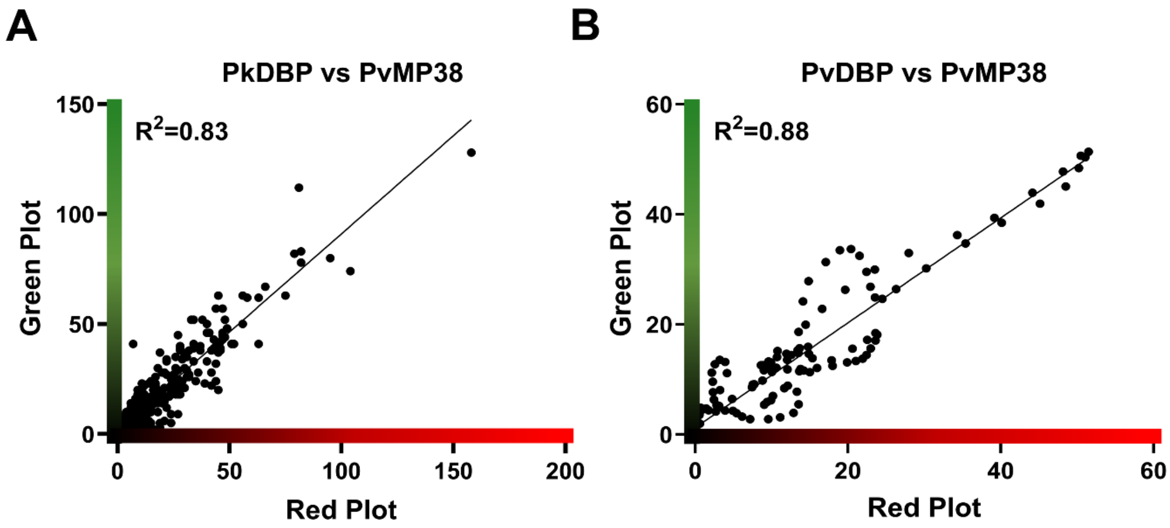

FIG S4. PvMP38 co-localizes with *P. vivax* and wild-type *P. knowlesi* DBP polyclonal antibodies. Mouse anti-PvMP38 (red) was dual labeled with rabbit immune sera against DBP (green), a localization marker. The correlations between green and red blot from the IFA image were calculated using Spearman's correlation test, and the R^2 value represented the overlapped strength.

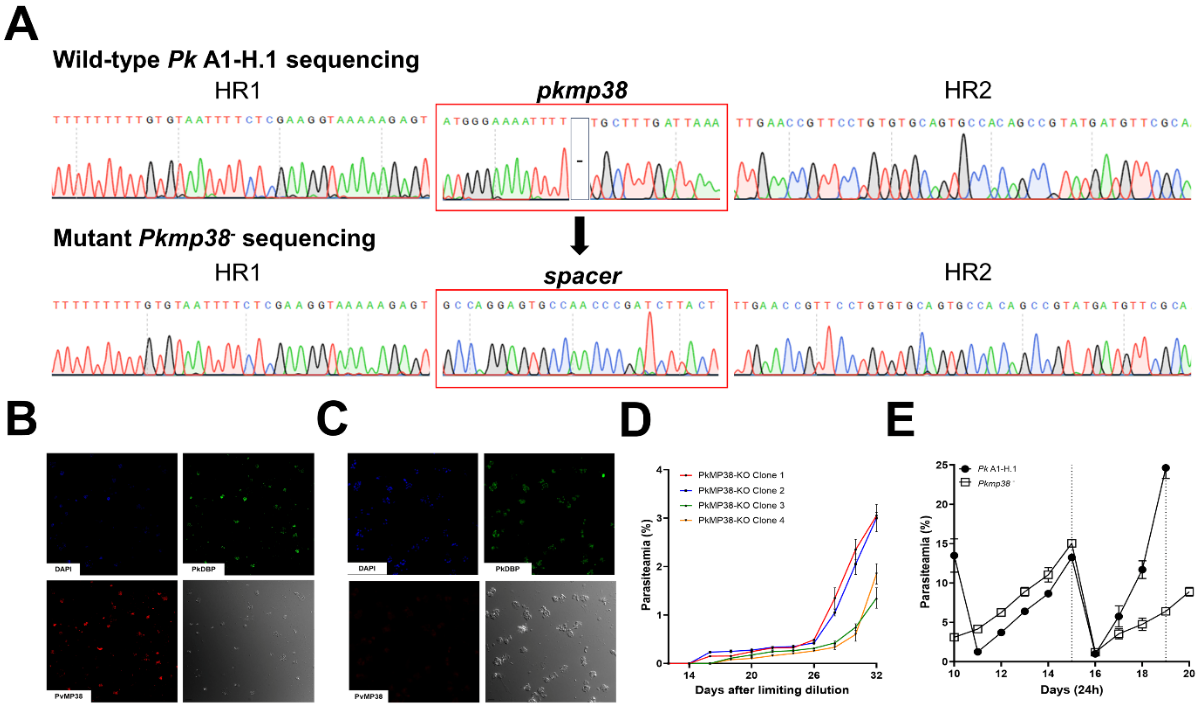

FIG S5. The *Pkmp38*$^{-}$ parasite could compensate for the deleted gene. (A) Locus sequencing of wild-type and mutant *Pkmp38*$^{-}$ strains aligned to the *P. knowlesi* reference genome. The gap represented the abridged *pkmp38* sequence. Replacement of the *pkmp38* to the spacer sequence confirms deletion of this from the *P. knowlesi* genome. The co-localization of wild-type *Pk* A1-H.1 (B), and *Pkmp38*$^{-}$ parasite (C) using the PvMP38 polyclonal antibody in an enlarged capture area. (D) After limiting dilution, the *Pkmp38*$^{-}$ strain parasitemia was followed until the parasite recovered in four different clones of Fig 4E experiment. (E) The wild type and mutant parasites were continued to culture and supplied new blood after measuring 10 cycles in Fig 4E. The dashed lines show the subculture whenever the parasitemia is more than 10%.
