# Supplementary material for: A novel micronemal protein MP38 is involved in the invasion of merozoites into erythrocytes
Source: mBio. 2025 Apr 9;16(5):e03917-24. doi: 10.1128/mbio.03917-24 (PMC12077092; doi:10.1128/mbio.03917-24)
Supplement: Supplemental tables — Tables S1 to S9. [file mbio.03917-24-s0002.pdf]

Table S1. Location of peptides identified by LC-MS/MS analysis of SDS-PAGE gels

All regions covered by identified peptides of hypothetical protein PVX_110945 [*Plasmodium vivax*], XP_001608414.1, are shown in red.

1. Band 1

- Protein sequence coverage: 42%

1 MGKFSGLSIF YFVFLLNFCL YKNGVQGDDM ANETNPRVRS GPSNNDHSEQ
51 MTKKLNRRNG THQNVRTNAR HYHGAVAGST GTRNLGSK**LV** **TGNEEVR**GNS
101 TSSDQSAAAS SGSSNTIGGM LAR**IFLNALV** **KPAVNFVVGG** **KDPVFPLPSE**
151 **NKAGQEDAQP** **GQRQEQQQQQ** **QQQQLEESPQ** **ELRREEQQQV** **ITPQPSRVTP**
201 **PQQEKPVTLI** **DNK**MFKLERD QQAHTSMNDL REEVQEEVYD DGDDDHVDED
251 TNNNTEEDED EEIVYRYGSD EEAKEIGEEQ GFSSLLNDET IHMTHIK**EHT**
301 **DVDEEKEKVE** **DLVKYMTNSL** **QENDQLVNTL** **SGLSDDITMF** **LRK**

2. Band 2

- Protein sequence coverage: 20%

1 MGKFSGLSIF YFVFLLNFCL YKNGVQGDDM ANETNPRVRS GPSNNDHSEQ
51 MTKKLNRRNG THQNVRTNAR HYHGAVAGST GTRNLGSK**LV** **TGNEEVR**GNS
101 TSSDQSAAAS SGSSNTIGGM LARIFLNALV KPAVNFVVGG K**DPVFPLPSE**
151 **NK**AGQEDAQP GQR**QEQQQQQ** **QQQQLEESPQ** **ELRREEQQQV** **ITPQPSRVTP**
201 **PQQEKPVTLI** **DNK**MFKLERD QQAHTSMNDL REEVQEEVYD DGDDDHVDED
251 TNNNTEEDED EEIVYRYGSD EEAKEIGEEQ GFSSLLNDET IHMTHIKEHT
301 DVDEEKEKVE DLVKYMTNSL QENDQLVNTL SGLSDDITMF LRK

3. Band 3

- Protein sequence coverage: 10%

1 MGKFSGLSIF YFVFLLNFCL YKNGVQGDDM ANETNPRVRS GPSNNDHSEQ
51 MTKKLNRRNG THQNVRTNAR HYHGAVAGST GTRNLGSKLV TGNEEVRGNS
101 TSSDQSAAAS SGSSNTIGGM LARIFLNALV KPAVNFVVGG KDPVFPLPSE
151 NKAGQEDAQP GQRQEQQQQQ QQQQLEESPQ ELR**REEQQQV** **ITPQPSRVTP**
201 **PQQEKPVTLI** **DNK**MFKLERD QQAHTSMNDL REEVQEEVYD DGDDDHVDED
251 TNNNTEEDED EEIVYRYGSD EEAKEIGEEQ GFSSLLNDET IHMTHIKEHT
301 DVDEEKEKVE DLVKYMTNSL QENDQLVNTL SGLSDDITMF LRK

Table S2. Binding kinetics of the protein-protein interaction measured by BLI assay

Binding Pair	Exp.	K_D (M)	K_{on} (M^{-1} s^{-1})	K_{off} (S^{-1})	Full X^2	Full R^2	Binding model
**hDARC-Fc & PvDBP-RII (194-521 aa.)-8His**	1	3.00×10^{-9}	2.25×10^{5}	6.78×10^{-4}	2.44	0.994	1:1
	2	2.39×10^{-9}	5.22×10^{4}	1.25×10^{-4}	2.004	0.999	
**Pv12-Fc & PvMP38-8His**	1	2.56×10^{-9}	1.52×10^{4}	3.89×10^{-5}	1.059	0.999	
	2	2.39×10^{-9}	5.22×10^{4}	1.25×10^{-4}	2.004	0.999	
**Pk12-Fc & PvMP38-8His**	1	8.33×10^{-10}	2.99×10^{6}	2.49×10^{-3}	0.734	0.998	
	2	9.60×10^{-10}	5.49×10^{4}	5.27×10^{-5}	3.481	0.998	
**Pv12-Fc & Pv41-8His**	1	1.22×10^{-8}	5.43×10^{4}	6.62×10^{-4}	1.163	0.998	
	2	1.61×10^{-8}	5.00×10^{4}	8.03×10^{-4}	0.777	0.998	
**Pk12-Fc & Pv41-8His**	1	1.90×10^{-8}	8.04×10^{4}	1.53×10^{-3}	0.681	0.997	
	2	1.14×10^{-8}	5.49×10^{6}	6.24×10^{-2}	8.001	0.995	
**Pv41-Fc & Pv12-8His**	1	5.55×10^{-10}	3.14×10^{5}	1.74×10^{-4}	0.669	0.999	
	2	5.12×10^{-10}	3.05×10^{5}	1.56×10^{-4}	0.858	0.999	
**Pv41-Fc & Pk12-8His**	1	1.84×10^{-7}	2.63×10^{5}	4.85×10^{-2}	0.791	0.997	
	2	1.60×10^{-7}	2.84×10^{5}	4.54×10^{-2}	0.391	0.997	

Table S3. Primer combination for *pkmp38* gene knock-out parasites (A) donor DNA preparation. (B) Primers for Cas9 vector preparation. (C) Primer pairs for genotyping diagnosis

(A)

	Size of HR1 / HR2 (bp)	Primer HR1	Primer HR2	Primer HR1 + HR2
pkmp38-KO	798 / 776	ol001 + ol002	ol003 + ol004	ol003 + ol006

(B)

	Annealing primers			Colony PCR diagnosis primers			
	sgRNA-1	sgRNA-2	sgRNA-3	sgRNA-1	sgRNA-2	sgRNA-3	Band size (bp)
pCas9/sgRNA	ol011 + ol012	ol013 + ol014	ol015 + ol016	ol011 + ol017	ol013 + ol017	ol015 + ol017	169

(C)

	Diagnostic primers	Band size (bp)	Diagnostic primers	Band size (bp)	Diagnostic primers	Band size (bp)
	WT locus		Integration locus		House-keeping gene	
PkA1-H.1 and *pkmp38*-KO	ol005 + ol008	1248	ol007 + ol006	796	ol009 + ol010	1055

Table S4. Seropositivity of IgG response of normal and heat-treated PvMP38 in vivax and knowlesi patients and healthy individuals

Antigen	Samples	*n*	No. of samples		Sensitivity (%)[a]/ Specificity (%)[b]	95%CI[b]	Normalized MFI[c]	*p*-value[e]
			Positive	Negative				
Native PvMP38	*P. vivax*	72	37	35	51.4[a]/	39.31-63.35	1.08	0.006
	P. knowlesi	56	23	33	41.1[a]/	28.1-55.02	0.94	0.15
	Healthy individuals	32	2	30	93.75[b]	79.19-99.23	0.58	
Heat-treated PvMP38	*P. vivax*	72	51	21	70.8[a]/	58.93-80.95	2	<0.0001
	P. knowlesi	56	29	27	51.8[a]/	38.03-65.34	1.02	0.001
	Healthy individuals	32	1	31	96.88[b]	83.78-99.92	0.6	

[a] Seropositivity/seropositive rate: percentage of positive in malaria patient samples.

[b] Specificity/seronegative rate: percentage of negative in total healthy samples.

[c] Confidence intervals.

[d] Normalized MFI: mean fluorescence intensities were divided by a cut-off value plus 2 standard deviations above the mean fluorescence intensity of the malaria-naïve samples.

[e] Differences in the total IgG prevalence for each antigen between vivax patients and healthy individuals were calculated using a multi-variant one-way ANOVA, and Tukey's secondary test, $p<0.05$ is considered statistically significant.

Table S5. Baseline characteristics of 72 *P. vivax* (A) and 58 *P. knowlesi* (B) infected patients enrolled in the study

(A)

Variables	Values
Age	
Mean ± SD	35.49 ± 15.92
Median (25th-75th)	31 (23 – 47)
Sex(%)	
Male	54 (75)
Female	18 (25)
Parasitaemia (%)	
Mean ± SD	0.11 ± 0.14

(B)

**Variables**	**Values**
Age	
Mean ± SD	36.29 ± 17.97
Median (25th-75th)	33.5 (20.5 – 52.25)
Sex (%)	
Male	43 (73.14)
Female	15 (25.86)
Parasitaemia (%)	
Mean ± SD	2.50 ± 4.87

Table S6. Protein expression ectodomains in the present study.

Gene ID	Common name	Length (aa)	Expected size (kDa) His/ Fc-tag	Obtained size (kDa) His/Fc-tagged	Region expressed
PVX_113775	Pv12	316	38.6/67.8	39/73	F24-A339
PKNH_1137300	Pk12	300	41/66.2	45/75	F24-S344
PVX_000995	Pv41	363	41.7/73.3	50/76	E22-E384
PVX_110945	PvMP38	316	35.6	35-43-57	D28-K343
PVX_110810	PvDBP	328	38.7	39	D194-T521
NP_002027	hDARC	189	38.5	40	M1-P63
XP_018652834	GST	218	25.5/57.1	26/58	M1-K218

Table S7. Out-of-frame Score (A) and off-target gene of sgRNAs (B) for *pkmp38* in *P. knowlesi* genome

(A)

PlasmoDB ID	Guide	GC Contents (%)	Out-of-frame Score	Number of found targets
PKNH_0728800	GCCACGCTCACGGAAACGTT	40.0	60.2	1

(B)

gRNA ID	gRNA sequence (PAM "|NGG")	Total score	efficiency score	Efficiency score	Off-target hits
PkMP38_sgRNA1	GCTGTCCTTGGCGTCCCTGG|TGG	0. 38	0. 48	0. 86	0 | 0
PkMP38_sgRNA2	GCATTATCACGGGGCAGTGG|TGG	0. 35	0. 57	0. 75	0 | 0
PkMP38_sgRNA3	GCCACGCTCACGGAAACGTT|GGG	0. 35	0. 57	0. 75	0 | 0

Table S8. Primer sequences in the present study

No.	Name	Sequence (5'->3')	Remark
1	ol001 PkMP38 HR1 rev	GATCGGGTTGGCACTCCTGGCACTCTTTTTACCTTCGAGAAAATTACACAAAAAAAAAAAG	Preparation of donor DNA for *pkmp38* gene knock-out
2	ol002 PkMP38 HR1 n1 fwd	GCAAAAGTAGGGTTGTGAAGGACG	
3	ol003 PkMP38 HR2 n1 rev	GTCTAAACGTCAAAGCTACACGGG	
4	ol004 PkMP38 HR2 fwd	GCCAGGAGTGCCAACCCGATCTTACTTATTTTGGGGATTGGTGCTTTGATTAAATT	
5	ol005 PkMP38 HR1 n2 fwd	CAATTTTGGCACTCCTTTTCTTCGCTTC	
6	ol006 PkMP38 HR2 n2 rev	CTGGGAGAATTCCCTTTTAAGGTGC	
7	ol007 spacer fwd	CCAGGAGTGCCAACCCGATC	*pkmp38* gene detection by genotyping PCR
8	ol008 PkMP38 HR1 n2 rev	TGGTGGAAACACAGGACTCTC	
9	ol009 PkMTIP_ctag_F	CCCGGGGCGTTTTCGCGTATCTGCGCTTTTTC	
10	ol010 PkMTIP_ctag_R	CCTAGGGGACAATATATCCTCACAGAACAACTTG	
11	ol011 PkMP38 seed 1 fwd	**TTACAGTATATTATT**GCTGTCCTTGGCGTCCCTGG**GTTTTAGAGCTAGAA**	sgRNA-1 annealed sequence
12	ol012 PkMP38 seed 1 rev	**TTCTAGCTCTAAAAC**CCAGGGACGCCAAGGACAGC**AATAATATACTGTAA**	
13	ol013 PkMP38 seed 2 fwd	**TTACAGTATATTATT**GCATTATCACGGGGCAGTGG**GTTTTAGAGCTAGAA**	sgRNA-2 annealed sequence
14	ol014 PkMP38 seed 2 rev	**TTCTAGCTCTAAAAC**CCACTGCCCCGTGATAATGC**AATAATATACTGTAA**	
15	ol015 PkMP38 seed 3 fwd	**TTACAGTATATTATT**GCCACGCTCACGGAAACGTT**GTTTTAGAGCTAGAA**	sgRNA-3 annealed sequence
16	ol016 PkMP38 seed 3 rev	**TTCTAGCTCTAAAAC**AACGTTTCCGTGAGCGTGGC**AATAATATACTGTAA**	
17	ol017 sgRNA seq rev	CCGCCAAGTGCACTACGAGGA	sgRNA sequencing

Table S9. List of programs used for pDNA construct generation

**Program**	**Step**	**Temperature(℃)**	**Time**	**Cycle**
Annealing sgRNA				
	1	95	5 min	1
	2	95 (-1℃/cycle)	1 min	30
	3	65	30 min	1
	4	65 (-1℃/cycle)	1 min	40
	5	4	Hold	1
Re-construct plasmid for guide RNA				
Digestion	1	60	1 hour	
In-fusion	2	50	20 min	
	3	4	Hold	
Colony PCR check				
	1	95	10 min	1
	2	95	30 sec	
	3	58	1 min	35
	4	72	1-1.5* min	
	5	72	10 min	1
	6	4	Hold	1
Genotype check				
	1	95	5 min	1
	2	95	20 sec	
	3	58	20 sec	35
	4	72	1-1.5* min	
	5	72	10 min	1
	6	4	Hold	1

* Depend on target (1min/kb).
